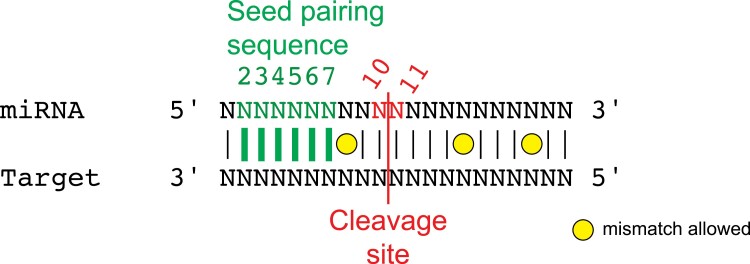# Using CRISPR/Cas to enhance gene expression for crop trait improvement by editing miRNA targets

**DOI:** 10.1093/jxb/erad003

**Published:** 2023-01-10

**Authors:** Savio S Ferreira, Rodrigo S Reis

**Affiliations:** Department of Biological Sciences and BioDiscovery Institute, University of North Texas, United States of America; Institute of Plant Sciences, University of Bern, Switzerland; Instituto de Agrobiotecnología del Litoral, Argentina

**Keywords:** CRISPR/Cas9, crop improvement, gene editing, miRNA, post-transcription

**Gene editing tools such as CRISPR/Cas9 are often thought of as a means to prevent gene expression; however, a more subtle and yet powerful approach is the enhancement of gene expression by precise deletion of repressor sites within a gene of interest. Recent reports demonstrate that editing sequences required for post-transcriptional regulation can result in transcript that is more stable or more translated, with consequent trait improvement in crop plants. Within this approach, gene editing of miRNA target sites is remarkably promising because of the typical conservation of miRNAs and their targets, and thus the likely ease with which the technique might be transferred from model to crop species. Here, we argue that gene editing of miRNA target sites is preferable over deletion of the miRNA genes themselves, given that it avoids the pitfalls of unspecific derepression of miRNA target(s) beyond the gene of interest.**

## Gene editing in crop improvement

Agricultural biotechnology is undergoing a revolution with the development of CRISPR/Cas gene-editing tools (Jinek *et al.*, 2012; Zaidi *et al.*, 2020). There have been numerous reports of CRISPR-mediated genome editing in economically important crops (Zaidi *et al.*, 2020), and cultivation and commercialization are already regulated in major world economies, such as in the United States, Canada, Brazil, Argentina, and Australia (Turnbull *et al.*, 2021). It is likely that in a few more decades, gene-edited food will be ubiquitous in most parts of the world.

Gene editing using CRISPR/Cas, in its simplest form, is based on two components that come together to form a ribonucleoprotein (RNP) complex that can specifically recognize and cleave a target site within the genome. Such an RNP complex is formed with an engineered non-coding RNA (i.e. the clustered regularly interspaced short palindromic repeat; CRISPR) and a Cas protein (Jinek *et al.*, 2012). Today, there is a vast diversity of CRISPR/Cas-based tools, as well as the use of different Cas proteins, that allow for a broad range of applications (Zaidi *et al.*, 2020). Due to restrictions on genetically modified crops (i.e. insertion of exogeneous genetic material), agricultural biotechnology has mostly benefited from CRISPR-mediated genome editing via transgenic expression of CRISPR/Cas followed by transgene removal through plant crossing and genetic segregation, as well as via ‘transgene-free’ approaches involving application of the RNP complex without plant genetic transformation (Chen *et al.*, 2019). For example, preassembled CRISPR/Cas RNPs using the Cas9 protein have been delivered using particle bombardment into maize and wheat embryos, and plants have been regenerated without the addition of exogenous genes (including no selection marker), resulting in on-target editing frequencies above 2% (Svitashev *et al.*, 2016; Liang *et al.*, 2017). In addition, new developments and optimization of these protocols and systems, such as the use of a miniature Cas protein that reduces the cargo to be delivered (Wu *et al.*, 2021), will probably facilitate their adoption in crop improvement. Although the editing efficiency is still not ­impressive and the technique is laborious, delivery of preassembled CRISPR/Cas RNPs is the ‘cleanest’ approach because no exogeneous DNA is ever introduced and, consequently, it is probably better perceived by the general public and be more likely to receive regulatory approval.

## Gene editing beyond knockout

Currently, most edited crops have been engineered for gene knockout (i.e. loss of function); however, the advantages of using more subtle approaches are becoming apparent. Gene knockout using CRISPR/Cas9 has enabled crop trait improvement such as increased yield, improved quality and stress resistance, and acceleration of breeding (hybrid breeding) (Chen *et al.*, 2019). Although as yet poorly explored, genome editing to modulate gene expression, as opposed to drastic inhibition, can enable fine-tuning of expression levels and distribution. For example, editing of regulatory genomic regions outside the gene of interest (e.g. *cis*-regulatory elements, such as the promoter region) has been done in tomato (Rodríguez-Leal *et al.*, 2017) and rice (Cui *et al.*, 2020), leading to altered gene expression and phenotypical alterations. Advantages of this approach include the possibility to modify gene expression in a tissue-specific or developmental stage-specific manner, as well as to manipulate gene expression responses to internal or external stimuli (Zhu *et al.*, 2020). When the introduction of an exogenous gene is not undesirable, a catalytically inactive Cas enzyme (dCas) fused to transcriptional activators or repressors can be used to regulate gene expression via sequence-specific recruitment (provided by the CRISPR sequence, i.e., the guide RNA) of the dCas–transcriptional regulator fusion to a specific promoter (Pan *et al.*, 2021).

Manipulation of post-transcriptional regulation using gene editing enables enhancement of gene expression, usually via the removal of inhibitory elements that limit expression. Such inhibitory elements can be found within transcripts (e.g. sequence and structural features, and binding sites) and proteins (e.g. modification and catalytic sites, and interacting regions) (Wray, 2003; Roy and Arnim, 2013; Liu *et al.*, 2016). Until recently, genome editing of post-transcriptional regulation applied to crop improvement has been limited to translational enhancement via targeting of upstream open reading frames (uORFs). uORFs are an example of a transcript sequence feature in which a sequence upstream the main ORF encodes for a peptide, usually not conserved, and such unproductive translation reduces efficiency or inhibits ribosomes from translating the main ORF. uORFs are commonly found in transcripts and editing of their start codon, as shown for the *LsGGP2* uORF using CRISPR/Cas9 in lettuce (*Lactuca sativa*) (Zhang *et al.*, 2018), can result in enhancement of the translation of the main ORF with a corresponding trait improvement (an increase in ascorbate content in lettuce in the example of *LsGGP2*). Sweeter strawberries (*Fragaria vesca*) have also been produced by enhancement of translation via uORF gene editing (Xing *et al.*, 2020).

**Fig. 1.**
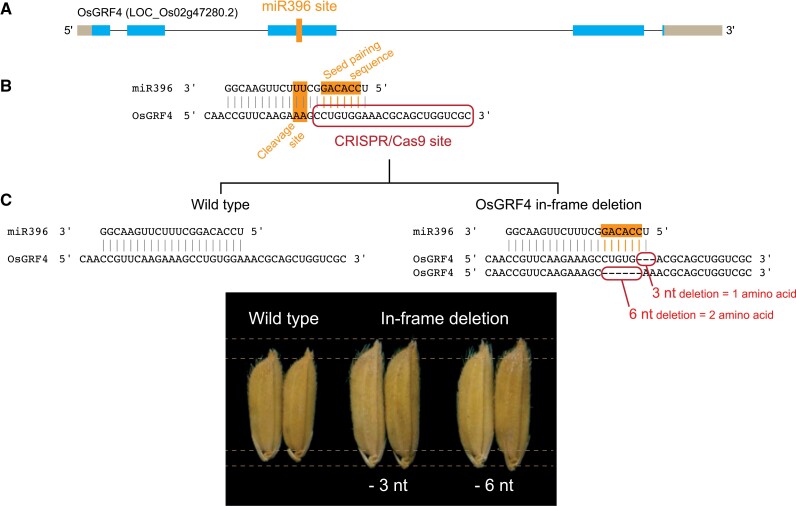
CRISPR/Cas9 deletion of the miR396 target site in *OsGRF4*. (A) The position of the miR396 target site in the *OsGRF4* locus. (B) Detailed miR396 target site in the *OsGRF4* transcript, highlighting the miRNA seed-pairing sequence and the transcript cleavage site. The illustration of the CRISPR/Cas9 target sequence is adapted from Lin *et al.* (2021). (C) Comparison of transcripts from the wild type and *OsGRF4* in-frame deletions obtained by Lin *et al.* (2021). (B) and (C) show RNA nucleotide sequences and the CRISPR/Cas9 target site is shown based on the corresponding genomic locus.

In plants, the stability and translation of several transcripts are tightly regulated by specific miRNAs that display sequence complementarity to binding sites within target transcripts. miRNA binding sites and the miRNAs themselves are usually conserved, in contrast to other inhibitory elements such as uORFs. miRNAs have also been targets of gene editing, and several reports have shown that CRISPR/Cas can be used to knockout specific miRNAs (recently reviewed by Deng *et al.*, 2022), thus enhancing target transcript gene expression with consequent trait improvement. However, this approach can result in pleiotropic or undesirable phenotypes because all targets of the knocked-out miRNA will potentially be released from its inhibition. An alternative approach that is likely to be more specific has recently been shown for rice (*Oryza sativa*) improvement (Lin *et al.*, 2021). The authors demonstrated that in-frame deletion of the miR396 target site within the *GROWTH REGULATING FACTOR 4* (*OsGRF4)* and *OsGRF8* coding sequences leads to enhanced transcript stability with consequent trait improvement (Fig. 1), with larger grains and improved resistance to brown planthopper, respectively. Interestingly, even one single nucleotide deletion within the miRNA seed pairing has been shown to effectively release a transcript from miRNA inhibition (Box 1). Indeed, targeting the seed region is possibly the most robust approach to eliminate a miRNA repression, because although deletion of the miRNA cleavage site is also likely to affect miRNA repression, it might allow for residual miRNA translation inhibition to occur, given that the deletion of the site does not prevent binding of miRNA-loaded argonaute complex (Box 1). In cases where in-frame deletion is detrimental to gene function, base editors and prime editors can be applied to introduce silent point-mutations, without the introduction or removal of nucleotides, to rewrite the target sequence and, consequently, alter its regulation by a specific miRNA. Base editors have been used to introduce a point-mutation in miR156 targets in rice, but no evidence has been shown for altered gene expression (Hua *et al.*, 2019). Another possibility is when the target site is at an untranslated region (UTR), where editing has more flexibility. For example, the target site for miR156 at the 3ʹ-UTR of *SQUAMOSA promoter-binding protein-like 13* (*TaSPL13*) in wheat has been edited using CRISPR/Cas9, producing different indels and resulting in a 2-fold increase in gene expression (Gupta *et al.*, 2023). This tipped the trade-off between growth and yield in favour of increased yield, with the edited plants producing more and bigger grains. However, this strategy is limited to a single gene in the *SPL* family, because the target sequence for miR156 is found in the coding sequence in all transcripts except for that of *TaSPL13*. As the CRISPR/Cas technology matures, gene editing will become more precise and predictable, for example with tools such as APOBEC-Cas9 (Wang *et al.*, 2020), and hence broadening the miRNA regulatory networks amenable to gene editing. Gene editing applied to post-transcriptional regulation, including miRNA inhibition, is a powerful strategy in crop improvement that is still poorly explored.

## Next steps in gene editing of post-transcriptional regulation

It is increasingly apparent that the path from gene to protein to function is far from orthogonal, given the myriad of regulatory mechanisms that modulates the abundance and activity of virtually all molecules found in a living organism. During transcription, modulations of processes that define the mature transcript sequence, such as transcription start-site usage, the polyadenylation signal, and alternative splicing, are commonly associated with the presence of regulatory elements, without necessarily altering the encoded amino acid sequence. For instance, differential transcript start-site usage modulates the presence of uORFs in response to light in the model plant Arabidopsis by producing transcripts with a shorter 5ʹ-UTR lacking inhibitory uORFs, thus exhibiting enhanced translation (Kurihara *et al.*, 2018). In fact, many regulatory processes previously thought to be independent from transcription occur co-transcriptionally, including RNA folding and RNA modification (Garcia-Lopez *et al.*, 2018; Huang *et al.*, 2019), blurring the line between transcriptional and post-transcriptional regulation. Once mature transcripts are released from the transcription site, regulatory elements found both in the transcript sequence and structure play essential roles in delivering the genetic information, as well as in the regulation of other processes, such as RNA-dependent complex formation and phase separation. During translation, the mRNA sequence might harbour distinct regulatory elements (e.g. the uORF and miRNA target site), as well as less understood or unknown features that modulate RNA stability or translation efficiency, including particular RNA structures. Once translated, protein function is often tightly regulated through residue modifications (e.g. phosphorylation) and specific interactions (e.g. with proteins and metabolites). Furthermore, the products of enzymatic activities can potentially vary with changes in enzyme composition, such as variation in the activity site. Although the list is far more exhaustive, these examples of various steps in which specific changes in the genome can potentially drive different biological functions illustrate the broad and largely untapped diversity of CRISPR/Cas9 targets for crop improvement.

Box 1. **Key elements in miRNA-target transcript base pairing and cleavage**

Most plant miRNAs are specific to a limited number of target transcripts, usually defined by perfect base pairing between the miRNA seed region and the target transcript (Brodersen and Voinnet, 2009). However, imperfect seed pairing is allowed in certain miRNA–transcript pairs, where base pairing with the miRNA 3ʹ-end compensates for the weak seed pairing. For instance, seed pairing in Arabidopsis miR398a–CDS2 exhibits one mismatch, while the remaining nucleotides exhibit only two mismatches.

Plant miRNAs guide transcript cleavage between their nucleotides 10 and 11. Therefore, a mismatch in the cleavage site abolishes transcript cleavage, which is usually readily quantified by RNA sequencing and quantitative PCR. However, a cleavage-impaired miRNA–argonaute complex might still drive inhibition of translation (Dugas and Bartel, 2008), and because translation regulation is rarely analysed in most studies, phenotypical results from plants lacking perfect base pairing within the cleavage site (e.g. via gene editing using CRISPR/Cas9) should be treated with caution.